# Reliability of X7R Multilayer Ceramic Capacitors During High Accelerated Life Testing (HALT)

**DOI:** 10.3390/ma11101900

**Published:** 2018-10-04

**Authors:** Ana María Hernández-López, Juan Antonio Aguilar-Garib, Sophie Guillemet-Fritsch, Roman Nava-Quintero, Pascal Dufour, Christophe Tenailleau, Bernard Durand, Zarel Valdez-Nava

**Affiliations:** 1 Universidad Autónoma de Nuevo León, CICFIM, San Nicolás de los Garza, N.L., MX 66455, Mexico; ahernandez@laplace.univ-tlse.fr; 2 CIRIMAT, Université de Toulouse, CNRS, INPT, UPS, 31062 Toulouse CEDEX 9, France; guillem@chimie.ups-tlse.fr (S.G.-F.); dufour@chimie.ups-tlse.fr (P.D.); tenailleau@chimie.ups-tlse.fr (C.T.); bdurand@chimie.ups-tlse.fr (B.D.); 3 LAPLACE, Université de Toulouse, CNRS, INPT, UPS, 31062 Toulouse CEDEX 9, France; 4 Universidad Autónoma de Nuevo León, FIME, San Nicolás de los Garza, N.L., MX 66455, Mexico; juan.aguilargb@uanl.edu.mx; 5 Kemet Charged (México), Antiguo camino al Mezquital 100, San Nicolás de los Garza, N.L., MX 66490, Mexico; romannava@kemet.com

**Keywords:** MLCC reliability, HALT, Y-doped BaTiO_3_, accelerated aging, X7R, activation energy

## Abstract

Multilayer ceramic capacitors (MLCC) are essential components for determining the reliability of electronic components in terms of time to failure. It is known that the reliability of MLCCs depends on their composition, processing, and operating conditions. In this present work, we analyzed the lifetime of three similar X7R type MLCCs based on BaTiO_3_ by conducting High Accelerated Life Tests (HALT) at temperatures up to 200 °C at 400 V and 600 V. The results were adjusted to an Arrhenius equation, which is a function of the activation energy (*E*_a_) and a voltage stress exponent (*n*), in order to predict their time to failure. The values of *E*_a_ are in the range of 1–1.45 eV, which has been reported for the thermal failure and dielectric wear out of BaTiO_3_-based dielectric capacitors. The stress voltage exponent value was in the range of 4–5. Although the *E*_a_ can be associated with a failure mechanism, *n* only gives an indication of the effect of voltage in the tests. It was possible to associate those values with each type of tested MLCC so that their expected life could be estimated in the range of 400–600 V.

## 1. Introduction

Multilayer ceramic capacitors (MLCC) are one of the most used passive components in consumer electronics. The global production of MLCCs accounts for about 3 trillion units per year [1] and its cost per capacitance is relatively low (around 0.1 USD per unit). They are considered to be reliable components in the design of consumer electronics. MLCCs can be fabricated with a base metal electrode technology (BME) [2], where successive layers of dielectric and electrodes are stacked and sintered to obtain an interdigitated electrode structure. The main dielectric material is based on ferroelectric barium titanate (BaTiO_3_).

The electromagnetic properties of BaTiO_3_ make it suitable as a base material for dielectric formulation for MLCC [3,4]. These capacitors maintain stable properties under their design conditions during their lifetime. Doping remains the strategy to adapt the dielectric properties of the dielectric layer to the target application, especially for broad temperature use under high electric fields [5,6].

Since reliability is a critical parameter in the design of electronic components, manufacturers keep track of it through different parameters. An accurate assessment of the reliability can be achieved through the periodic analysis of products on service and by accounting for failed products, but this is not predictive and takes a long time. Therefore, other methods are required to reduce the evaluation time, which should also be able to provide an indication of the reliability prior to the commercial release of the components. Accelerated test conditions must be designed to obtain the most accurate prediction possible of the lifetime of capacitors under service conditions. The most common tests for the reliability of MLCCs are the insulation resistance and combined stress measurements, which is also known as the Highly Accelerated Life Test (HALT) [7].

HALT uses the simultaneous application of an electric and thermal stress while measuring the time to failure (TTF) for the MLCCs. These two combined factors can induce a shorter time to failure that provides information about the expected life under service conditions. In the case of the temperature, as for other thermally activated processes, failure seems to follow an Arrhenius-type behavior (Equation (1)), with the failure frequency (inverse of time to failure) increasing with an increase in temperature [8,9,10].

(1) $$\;\frac{1}{t}=Ae^{-E_{a}/kT}\;$$

*t*: time to failure (TTF) (s);
*E_a_*: activation energy for the thermally activated process (eV);
*k*: Boltzmann’s constant (8.62 × 10^−5^ eV/K);
*T*: absolute temperature (K);
*A*: pre-exponential factor (s^−1^).

The TTF due to thermal activation at a given temperature can be calculated from the data at different temperatures if the activation energy is known from Equation (1), as a set of two time-temperature conditions (Equation (2)).

(2) $$\;\frac{t_{1}}{t_{2}}=e^{\frac{E_{a}}{k}\left(\frac{1}{T_{1}}-\frac{1}{T_{2}}\right)}\;$$

where *t*_1_ = TTF, at *T*_1_ and *V*_1_, (s); and *t*_2_ = TTF, at *T*_2_ and *V*_2_, (s).

In Equation (2), both the activation energy and the pre-exponential factors in Equation (1) are constant. Regarding factor *A*, it represents the minimum TTF, which is consistent with the fact that these processes do not take place instantaneously, even at a very high temperature.

Since the electric field affects the time to failure of the capacitors, an empirical equation in which the pre-exponential factor that is still constant is modified by the applied voltage, has been proposed (Equation (3)) [6].

(3) $$\;\frac{t_{1}}{t_{2}}={\left(\frac{V_{2}}{V_{1}}\right)}^{n}e^{\frac{E_{a}}{k}\left(\frac{1}{T_{1}}-\frac{1}{T_{2}}\right)}\;$$

*t*_1_ = TTF, at *T*_1_ and *V*_1_, (s);
*t*_2_ = TTF, at *T*_2_ and *V*_2_, (s);
*V*_1_ and *V*_2_ = test voltages under conditions (V);
*n* = voltage stress exponent;
*E*_a_ = activation energy for dielectric wear out (eV);
*k* = Boltzmann’s constant (8.62 × 10^−5^ eV/K);
*T*_1_ and *T*_2_ = absolute test temperature (K).

These factors are well-described in the literature [6] and the expected coefficient of the voltage stress exponent (*n*) is in the range of 1.5–7.1 [6,11,12,13]. This great variation in *n* is an indicator of the uncertainty related to the effect of the voltage. It also highlights the importance of determining any correlations of the basic structural and chemical formulation with the reliability (time to failure) of capacitors.

Very few studies in the literature addressing this were found, which is understandable because producing MLCCs for reliability testing requires at least pilot size facilities to produce many samples with a reproducible process in order to minimize the influence of other parameters than the one being tested.

Moreover, the dielectric material is designed according to the requirements for a fixed type of capacitor and studying the effect of the doping chemistry on the reliability of the MLCCs is a relatively complex task.

Additives and dopants include cations, such as Mn^2+^, Mg^2+^, and Ca^2+^, which can partially compensate for the electrons and holes that the system might have due to the presence of oxygen vacancies [5,14]. They also include sintering aids, such as SiO_2_, which reduce the sintering temperature. Indeed, it has been reported that SiO_2_ leads to the formation of a liquid phase from the ternary system BaO-TiO_2_-SiO_2_, which reduces the eutectic point from 1320 °C to near 1260 °C [15,16]. Dy^3+^, Ho^3+^, Sm^3+^, La^3+^, Yb^3+^, or Y^3+^ are intended to substitute Ba and Ti cations in the BaTiO_3_ structure [17,18], but Dy^3+^, Ho^3+^, and Y^3+^ can also exhibit amphoteric behavior (occupying A- or B-sites) and are described as being helpful in prolonging the lifetime of the MLCCs [3]. Y_2_O_3_ is commonly employed as a dopant in the commercial formulation of powders for the fabrication of MLCCs because at an industrial scale, it results in similar properties compared to adding Ho_2_O_3_, Er_2_O_3_, or Dy_2_O_3_, and it is less expensive [17]. The presence of Ca^2+^ has been related to an improvement in the reliability of MLCCs. 

Given the numerous and complex interactions between the dopants during and after the sintering process, a simpler approach needs to be undertaken. In this work, we determined the time to failure of BaTiO_3_-based X7R (classification according to the Electronics Components, Assemblies & Materials Association, Arlington, VA, USA) MLCC capacitors containing 1.05 wt% yttrium and a certain amount of calcium in the base formulation.

## 2. Materials and Methods

Three different formulations were prepared using commercial grade barium titanate powders (Table 1). They were mixed with an organic media (acrylic polymers) to produce a 7.5 µm thick layer by slip casting.

**Figure 1:**
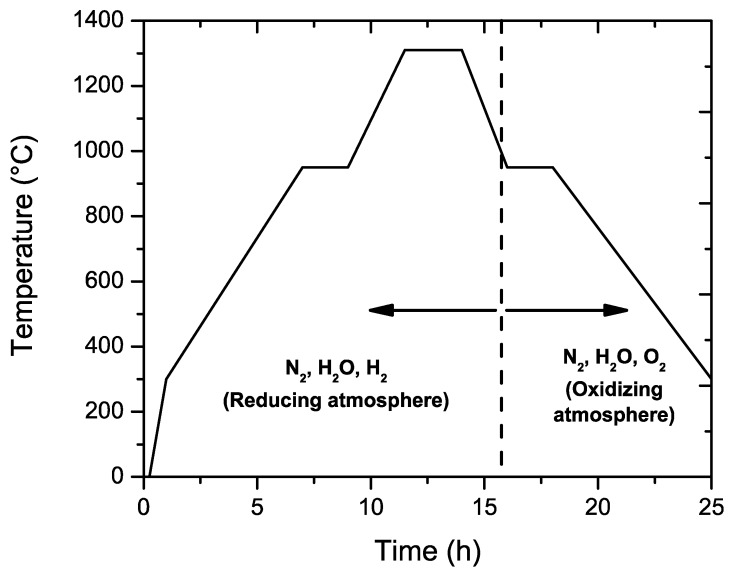
Sintering profile for the BaTiO_3_-based MLCCs for this study. Sintering is carried out in a reducing atmosphere and subsequent annealing is performed in an oxidizing atmosphere.

On the dried ceramic tape, a layer of nickel paste was deposited by screen printing to form the metallic electrodes. A total of 68 layers were pressed together, before being cut into slices with dimensions of 17 × 16 cm before the final shaping to form the green capacitors (1.8 mm length, 0.8 mm × 0.8 mm of cross-section). Sintering was carried out in a reducing atmosphere (mixture of H_2_/H_2_O with PO_2_ < 10^−8^) in order to avoid the oxidation of the nickel electrodes, before the oxidation step in air at lower temperatures (≈ 900 °C re-oxidation). This atmosphere prevents the oxidation of the Ni electrodes, but also affects the oxygen vacancy concentration. The full thermal treatment (sintering + re-oxidation) cycle is presented in Figure 1.

### 2.1. Multilayer Ceramic Capacitors Preparation

Surface polishing was carried out to expose the electrodes after sintering. To achieve electrical contact with the interdigitated electrodes, a copper cap was applied to the capacitors with an Ni layer and Sn termination. All the processes followed the standard handling processes of a pilot-scale line at Kemet (Kemet Charged, San Nicolás de los Garza, Mexico) facilities.

The samples were collected randomly from a batch of several thousands of capacitors sintered for each composition in order to perform the electrical measurements.

### 2.2. Electrical Characterization

**Figure 2:**
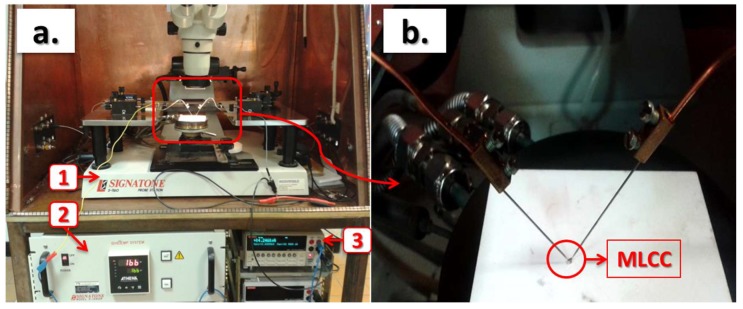
(**a**) Experimental set-up: (1) probe station, Signatone S-1160; (2) heating chuck, Signatone S-1060R; (3) sourcemeter unit, Keithley 2410. (**b**) Enlarged view of the MLCC under test.

After measuring the initial value of capacitance and dielectric losses (tanδ), which was in the range of 10^−2^ Hz to 10^6^ Hz with an applied voltage of 0.1 V_rms_ (Novocontrol Alpha-A, GmbH, Montabaur, Germany), the time to failure was measured under combined stress conditions. A voltage of 400 V and a temperature of 140 °C were applied to the sample. Two setups were used. First, an individual sample was tested on a thermally regulated probe station (Figure 2). In this case, the contacts with the sample are ensured with needle micromanipulators. A source meter unit (SMU 2410, Keithley, Cleveland, OH, USA) was used to apply the voltage and measure the current and time during the test. A fast rise in the current, which means a decrease in the resistivity to be near zero, indicated the moment when the dielectric breakdown of the capacitor occurred. 

**Figure 3:**
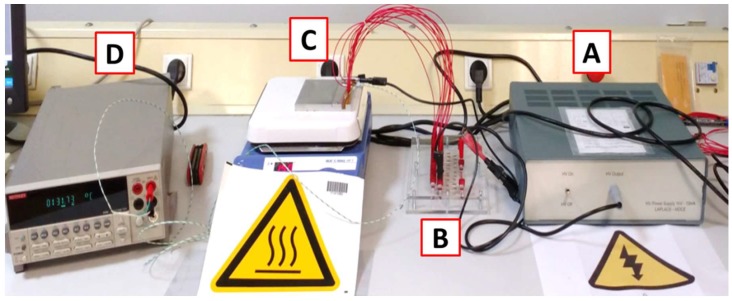
System for performing HALT on multiple samples at once. (**A**) HV power supplier (1 kV–10 mA); (**B**) Plate with protective resistors in parallel to connect the samples that are inside (**C**), a metallic cell placed over a heating plate, the temperature of which is monitored with (**D**), a voltmeter reading the measurements of a thermocouple (K-type).

With the purpose of testing multiple samples while controlling the experimental conditions, a second setup was developed and built in-house (Figure 3). A voltage source (1 kV–10 mA) (MPS1P10/24, Spellman, Hauppauge, NY, USA) (Figure 3A) was connected to a parallel array (Figure 3B) of capacitors placed inside a thermally stabilized hotplate (Figure 3C), and the temperature was measured with a thermocouple and kept at a uniform value (Figure 3D).

**Figure 4:**
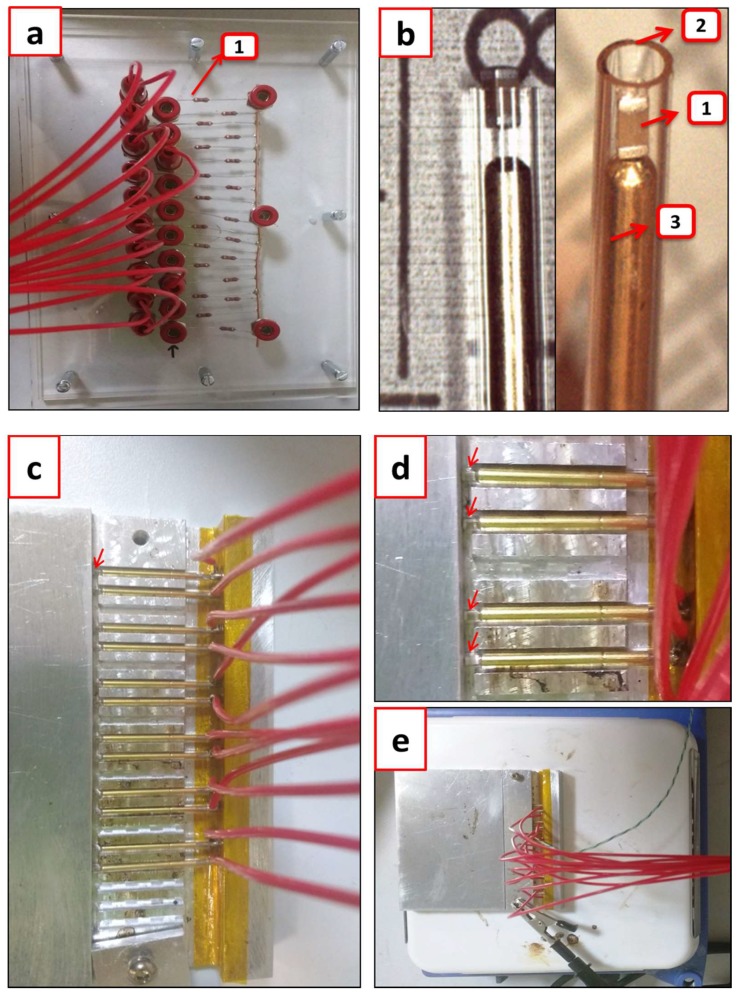
(**a**) Plate with the protective resistors (1) connected in series with each sample; (**b**) View of an MLCC (1) inside the glass capillary (2) together with a spring (3); (**c**) View of the assembled connections (cables + springs) together with the MLCCs in the metallic plate interior canals; (**d**) Enlarged view of the interior of the cell, where red arrows highlight the samples inside of a glass capillary; (**e**) View of the closed cell (with the samples inside) above the heating plate ready for the test.

The setting up of the samples for the test performance includes the details shown in Figure 4. To maintain the voltage after a capacitor failed, the protective resistors were joined (Figure 4a) and considering the total current that the dc supply could deliver (1 mA), the system had a maximum capacity of 20 samples. To connect the samples (Figure 4b-1) and avoid welding, they were placed inside a capillary glass (Figure 4b-2) and pressed with a spring test probe (Figure 4b-3), which ensured both the electrical contact and the mechanical support of the sample. The glass tubes with the samples and springs were placed inside an aluminum block to maintain thermal homogeneity (Figure 4c–e). The aluminum block also served as the electrical ground. The heating plate temperature was fixed and measured with a thermocouple (K-type), which was inserted inside a glass tube in the aluminum block. The temperature was always kept below 280 °C, which takes into account the melting temperature (296–300 °C) of the solder material used to assemble the springs with the connecting cables. When the set-up was ready and the desired temperature reached, the electrical stress was applied.

## 3. Results and discussion

**Figure 5:**
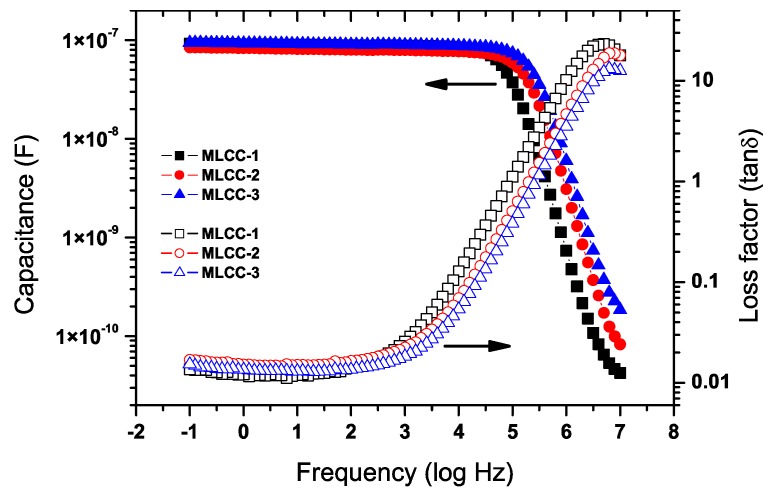
Capacitance and dielectric losses of the three types of MLCCs for this study. Capacitance values for all MLCC types are close to 100 nF.

**Figure 6:**
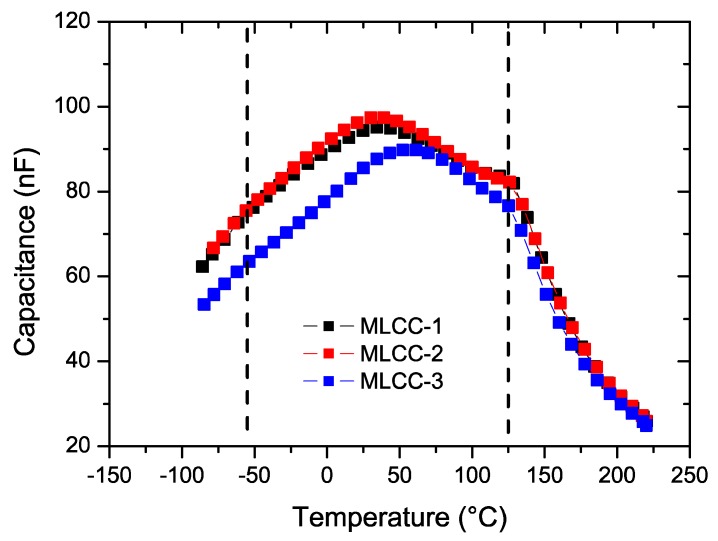
Capacitance of all three types of MLCC capacitors at 1 kHz. Dashed lines correspond to the X7R temperature specification limits.

The room temperature capacitance (Figure 5) was similar for the three compositions, which was found to be around 0.1 µF below 10^4^ Hz with similar dielectric losses. The X7R classification specifies that the capacity values should be within a range of ±15% when the capacitor is exposed to a temperature excursion between −55 °C to +125 °C. Figure 6 shows the impact of temperature on the capacity values at 1 kHz.

### 3.1. Initial HALT Test Results

**Figure 7:**
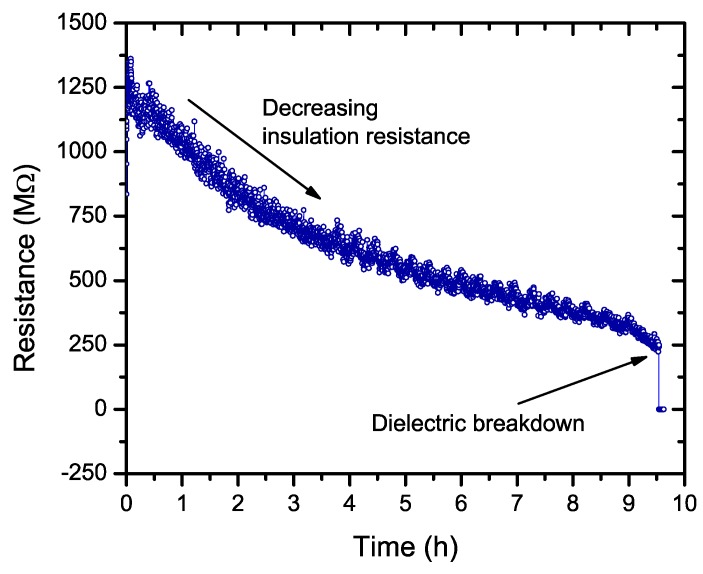
Insulating resistance decrease during individual sample tests under combined stress conditions (140 °C, 400 V) for MLCC-3 capacitors. The dielectric breakdown of the MLCC is detected when there is a sharp drop in the resistance value.

We first measured the time to failure under HALT conditions of 140 °C and 400V for the three compositions (Table 2). The main observation was that for the MLCC-2 composition, the time to failure was extremely short and it was practically impossible to apply a voltage after the samples were heated. In Figure 7, the current evolution is shown for the MLCC-3 sample during the individual tests. This increase in the current is also described as the insulation resistance of the MLCC and is often used as a complementary criterion for testing the samples [19].

**Figure 8:**
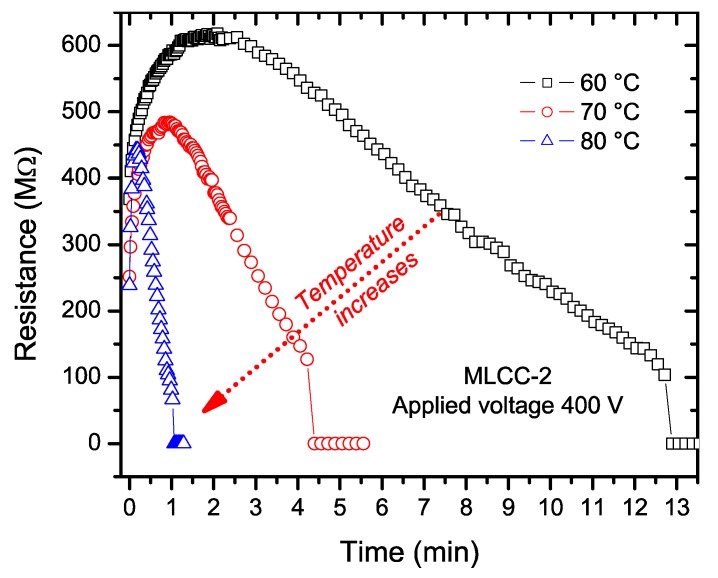
Change in the resistance value of an MLCC-2 at temperatures below 80 °C and 400 V. All three samples exhibit an initial increase in the resistance values, followed by a decrease, before breakdown.

We modified the temperature test conditions for the individual measurements of the insulating resistance of MLCC-2 samples and lowered the temperature to confirm that samples were not only failing due to the heating process. By lowering the temperature to 60 °C, we were able to observe a decrease in the resistance before the failure of the capacitors. The change in the resistance value for MLCC-2 was individually tested at temperatures below 80 °C, with the results shown in Figure 8.

Since the times to failure between MLCC-1 and MLCC-2 are extremely different, we performed complementary tests at different temperatures to evaluate the activation energies of the failure processes.

**Figure 9:**
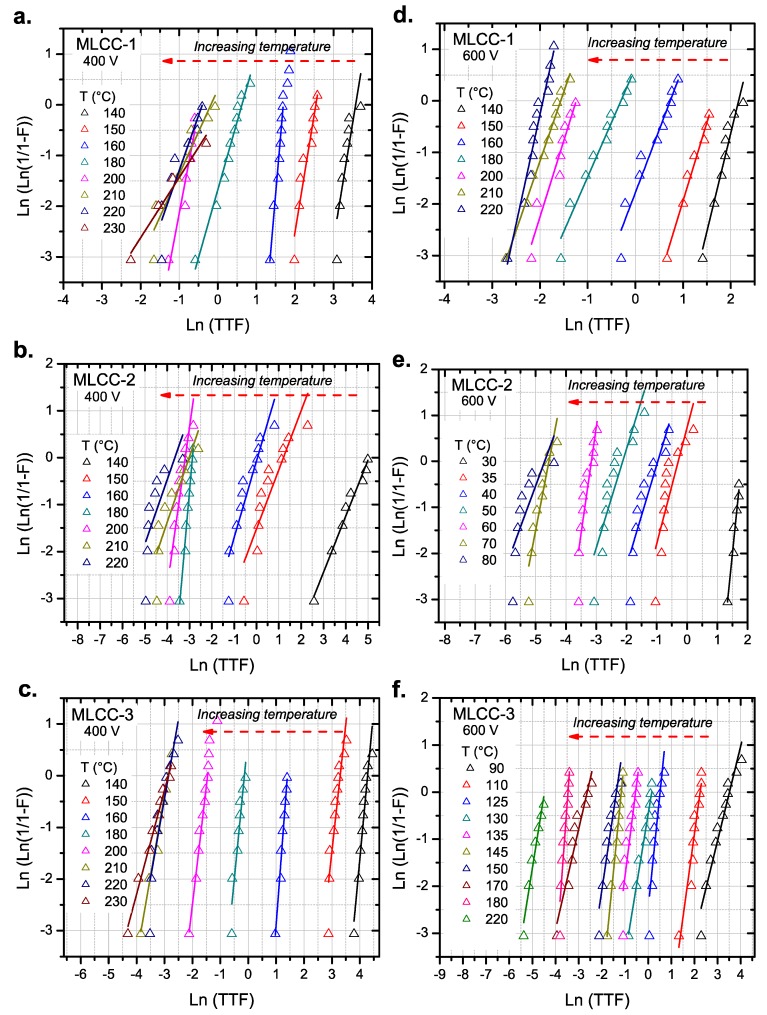
Weibull plot for MLCCs tested under a wide range of temperatures and different electrical stresses: (**a**), (**b**), and (**c**) 400 V; (**d**), (**e**), and (**f**) 600 V. Lines correspond to the linear fit used to determine the scale parameter (*α*) of the Weibull distribution. The increase of the temperature shifts the dielectric breakdown of the sample to lower times-to-failure.

The results for the parallel testing of the capacitors are shown in Figure 9. Since the time to failure follows statistical behavior, we plotted the results in a Weibull-type plot.

Measurements were performed at 400 V and 600 V to evaluate the impact of the voltage on the TTF of the MLCCs. 

**Figure 10:**
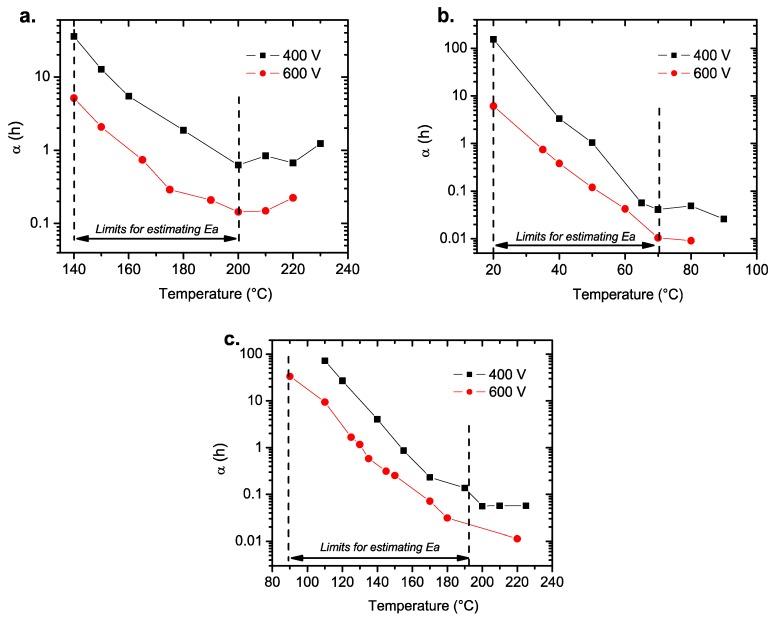
Changes in the scale parameter as a function of the temperature for: (**a**) MLCC-1, (**b**) MLCC-2, and (**c**) MLCC-3. The temperatures delimited by the dashed lines were considered for the Arrhenius plot in the next section.

Figure 10 shows the variation of the scale parameter (*α*) for all MLCC types at 400 and 600 V. For the three compositions, the high temperature data appear to shift away from the linear tendency observed at lower temperatures, which is seen in Figure 10a–c. This deviation in the behavior can be related to a different failure mode activated at high temperatures (T > 200 °C). 

### 3.2. Activation Energies

**Figure 11:**
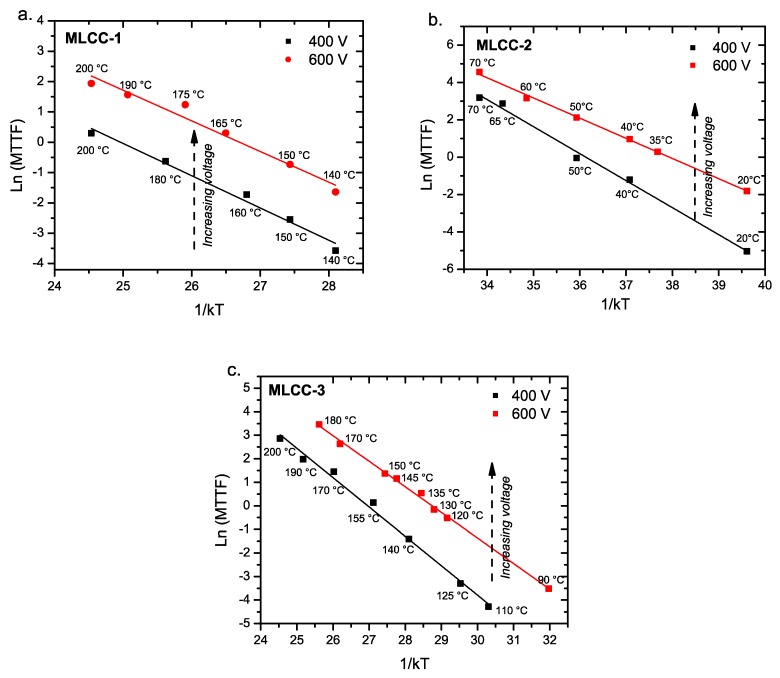
Characteristic life according to the Arrhenius model for (**a**) MLCC-1, (**b**) MLCC-2, and (**c**) MLCC-3, according to the electrical and thermal stress. Solid lines represent the best fits given by Equation (5).

As we plotted the data according to Equation (1), the activation energies can be estimated if we only consider the data below 200 °C for the MLCC-1 and MLCC-3 (Figure 10a,c) samples and below 70 °C for the MLCC-2 (Figure 10b) samples. Equation (1) shows the relationship of the frequency of failure (mean time to failure; MTTF), as shown below in Equation (4), which can be transformed into Equation (5) to obtain the activation energies related to the failures of the three types of MLCCs. The *E_a_* values were determined by applying the Arrhenius model to the life time of the capacitors (Figure 11).

(4) $$\;\frac{1}{t}=Ae^{-E_{a}/kT}=MTTF\;$$

(5) $$\;\ln\left(MTTF\right)=\ln\left(A\right)-\;\frac{E_{a}}{kT}\;$$

where MTTF is the mean time to failure (s^−1^).

The value of the *E*_a_ can be taken as a measure of the effect that the imposed thermal stress over the capacitor has on its life. Since the pre-exponential factor in this equation includes both the electrical stress and the thermal contributions (Equation (3)), a large activation energy value will imply a large effect of the imposed temperature on the capacitor life.

### 3.3. Discussion on Activation Energies

The activation energies are reported in Table 3. Those values are calculated by assuming that the voltage and thermal effects are a single combined factor and thus, the activation energy was calculated following the Arrhenius expression.

The numeric values of the activation energies should be related to the failure mechanism of the dielectric breakdown under stress conditions. It has been reported in general that the activation energy value of 1.9 eV was associated with an avalanche breakdown mode [20]; while this can be in a range of 1.25–1.42 eV for the thermal activated failure [21,22]. Other authors have suggested that for a thermal breakdown in MLCCs, the *E*_a_ will fall within the range of 1–2 eV [22]. Furthermore, the values in a range of 1.3–1.5 eV have been described as being related to dielectric wear in BaTiO_3_-based dielectric capacitors [23].

Even if the activation energy ranges overlap, this does not contribute to defining the exact failure mechanism. Among the possible mechanisms that may explain the obtained values, Chazono et al. [24] proposed that the oxygen vacancies migrate under the influence of the high electrical field that is applied during the HALT testing. Yoon et al. [23] suggested that as the vacancies are accumulated at the electrode interface, a Fowler-Nordheim (tunneling) conduction mechanism can be induced, which favors the breakdown of the dielectric layer due to the local increase in the conductivity. The activation energies within the range of 1–2 eV fall reasonably within the range of the *E*_a_ required for the mobility of oxygen vacancies [25,26]. In the present study, higher doping with calcium had a significant impact on the time to failure of the MLCC-2 samples.

A rough estimation of *n* for MLCC-1 composition, which only considered the test conducted at the same temperature, so that the relationship excludes the thermal part between 400 V and 600 V, gives the average value reported in Table 4 (*n* (*T*_1_ = *T*_2_)). To calculate this parameter with all the temperatures and the two voltages by means of the objective function (Equation (6)), the obtained values of n are shown in Table 4 ((*n* (all conditions)).

(6) $$\;fo=-\ln\left(\frac{t_{1}}{t_{2}}\right)+n\ln\left(\frac{V_{2}}{V_{1}}\right)+\frac{Ea}{k}\;\left(\frac{1}{T_{1}}-\frac{1}{T_{2}}\right)\;$$

Although all values in Table 4 seem to be consistent with other reports [22], the most accurate is *n* (all conditions) because it predicts the failure time under more conditions.

In the present study, higher doping with calcium had a significant impact on the time to failure of the MLCC-2 and MLCC-3 samples. Calcium has been reported as an acceptor doping element in BaTiO_3_. Although this can increase the overall resistivity of the ceramic [26], it can also alter the equilibrium of the oxygen vacancies. Moreover, the dopants can substitute Ba and Ti cations in the BaTiO_3_ [3]. Ca^2+^ (ionic radius ≈ 0.99 Å) tends to occupy the Ba-site, due to its size and valence, more than other dopants of the same valence. There are reports about calcium inducing a longer life span than other BaTiO_3_-based dielectrics, introduced alone in the structure [27] or in the presence of other elements (Dy_2_O_3_, MgO, MnO, and SiO_2_) [28]. Nakamura et al. [28] reported that the time to failure, as a function of Ca content, increased up to 8 mol%, remaining constant up to 12 mol%, and then decreased for further Ca content. The activation energies also followed a similar tendency, from 1.45 eV with no Ca added to 1.8 eV with 10 mol%, and then dropped to 1.5 eV at 12 and 14 mol%. It is speculated that this could be related to the segregation of CaTiO_3_ and that the observed activation energy corresponds to the electromigration of oxygen vacancies. If we assume that the necessary energy to activate the MLCC failure is the observed activation energy (*E*_a_), then the values for MLCC-2 samples are in contradiction to this assumption. This shifted our attention to the pre-exponential factor (*A*) in the Arrhenius equation. As we calculated the *A* values (Table 3), we noted that the greatest value (note that the values are in ln(*A*)) corresponded to the MLCC-2, hence compensating for the larger *E*_a_ in the MTTF calculation. The *A* value is considered as a frequency factor and the changes in the bonding energies within the structure, induced by doping, could also change its numerical value.

## 4. Conclusions

In the present work, we aimed to identify the impact of differences in the chemical composition of Y-doped BaTiO_3_ on the lifetime of MLCCs under accelerated conditions. Despite the apparent small differences in the chemical composition, the lifetime of the MLCCs can change from tens of hours (36 h) to less than a second at 400 V and 140 °C. The change in the Ca content induces a great drop in the lifetime from equivalent sintering and processing conditions. Despite this, only when thermally evaluated, the activation energies of the failure remain within the same range of 1–1.45 eV, which could be related to the activation energies of oxygen vacancies in BaTiO_3_. Although the thermally activated processes are well-described by the Arrhenius expression, the effect of the electric field is not as simple as the empirical equation claims. It can be suggested from the results that the presence of cations, which was Ca in this case, reduces the activation energy, which thermally reduces the life expectation, but increases the reliability of the MLCC in terms of sensitivity to the electric field. This is reflected not only in the activation energy (*E*_a_) and the voltage sensitivity exponent (*n*), but also through the pre-exponential factor (*A*) of the Arrhenius relationship. To further understand the failure mechanism of the MLCCs, the relationship between the microstructure and structural evolution during aging must be established, by observing the compositional evolution and how it is related to the Ca content in the BaTiO_3_, in addition to its role in the formation the secondary phases.

## Figures and Tables

**Table 1:** Chemical composition of the initial powders used in this study. Values are obtained by Inductively Coupled Plasma emission spectra (ICP) analysis. For all powders, the trace elements (Sr, Si, Mn, Mg, Co) account for less than 0.8 wt%.

Element	MLCC-1	MLCC-2	MLCC-3
Ba/Ti ratio	2.88	2.86	2.85
Y wt%	1.05	1.05	1.05
Ca wt%	< 0.01 (≈ 0.01 mol%)	0.5–0.6 (≈ 2.8 mol%)	1.3–1.5 (≈ 8.1 mol%)

**Table 2:** Time-to-breakdown for the MLCC at the initial HALT test conditions.

Sample Type	Time to Breakdown at 140 °C and 400 V
MLCC-1	37 h
MLCC-2	<1 s
MLCC-3	3.5 h

**Table 3:** Activation energy values obtained from the Arrhenius model for MLCCs tested under two different voltages and a range of temperatures (Figure 11).

MLCCs	*E*a (eV) at 400 V	*E*a (eV) at 600 V	ln(*A*) at 400 V	ln(*A*) at 600 V	Temperature Range at 400 V	Temperature Range at 600 V
MLCC-1	1.06 ± 0.07	1.01 ± 0.09	26.96 ± 2.5	26.52 ± 2.1	140–200 °C	120–200 °C
MLCC-2	1.45 ± 0.05	1.08 ± 0.02	39.90 ± 2.9	38.30 ± 1.8	20–70 °C	20–70 °C
MLCC-3	1.25 ± 0.06	1.09 ± 0.02	33.62 ± 1.3	31.23 ± 0.7	110–200 °C	90–180 °C

**Table 4:** Voltage stress constant (*n*).

MLCCs	*n* (*T_1_* = *T_2_*)	*n* (All Conditions)
MLCC-1	5.51	4.46
MLCC-2	4.95	3.26
MLCC-3	1.93	5.02
